# Deaths, Countermeasures, and Obedience: How Countries' Non-pharmaceutical Measures Have Quelled the COVID-19 Death Toll

**DOI:** 10.3389/fpubh.2022.934309

**Published:** 2022-06-24

**Authors:** Angelo Capodici, Davide Gori, Jacopo Lenzi

**Affiliations:** Department of Biomedical and Neuromotor Science, Alma Mater Studiorum – University of Bologna, Bologna, Italy

**Keywords:** excess mortality, lockdown, COVID-19, stringency index, mobility trends

## Introduction

From 2020 onwards, a steep increase in overall deaths was registered in many countries as a result of the COVID-19 pandemic. While many of these deaths were directly associated with the novel coronavirus disease, not all of them can be explained by it (1, 2).

To deal with this new biological threat, almost all the world's countries decided to enforce non-pharmaceutical interventions. These measures have been distilled by The Oxford Coronavirus Government Response Tracker (OxCGRT) into a single index, called “stringency index” (3), which includes school closures, workplace closures, cancellation of public events, restrictions on public gatherings, closures of public transport, stay-at-home requirements, public information campaigns, restrictions on internal movements, and international travel controls. Certainly, to be effective, restrictions need to be respected by citizens to appreciate their end results; however, stringency provisions were not always welcomed as salvific measures, and as fear of the disease thinned out, discontent of part of the population grew (4).

After more than 2 years since the World Health Organization declared COVID-19 as a full-fledged pandemic (5), our aim is to systematically evaluate how non-pharmaceutical measures impacted the pandemic trend. Although numerous variables could be examined, such as COVID-19 infections and deaths, excess mortality is a more comprehensive indicator that relies on all-cause mortality instead of specific causes of death (2), thus underlining how the whole healthcare system endured the pandemic blow.

In this study, we aimed to analyze to which extent the number and strictness of government policies in six countries [France, Germany, Italy, Spain, the United Kingdom (UK), and the United States (US)] reduced excess deaths associated with COVID-19 as well as peoples' mobility, used here as a proxy for compliance with COVID-19 restrictions.

## Methods

The data used in this time-trend analysis were gathered from Our World in Data (OWID) (6), one of the leading scientific online organizations publishing global data and research on the COVID-19 pandemic. Our analysis covered the period March 15, 2020 to March 20, 2022 (105 weeks), and was restricted to five countries of Europe (France, Germany, Italy, Spain, and the UK) and the US. These countries were selected to compare similarly structured healthcare systems: Beveridge-like for Italy, Spain, and the UK; Bismarck-like for Germany and France; a free-market type of healthcare system for the US. For each country, the following OWID variables were retrieved:

· Government stringency index, a composite measure based on nine response indicators including school closures, workplace closures, and travel bans, rescaled to a value from 0 to 100 (100 = strictest) (3).
· Excess mortality, measured as the cumulative difference between the reported number of deaths since January 1, 2020 and the projected number of deaths for the same period based on previous years, per million people. All-cause mortality data is from the Human Mortality Database Short-term Mortality Fluctuations project and the World Mortality Dataset. Both sources are updated weekly, the first date with available estimates for all study countries being March 15, 2020. The reported number might not count all deaths that occurred due to incomplete coverage and delays in reporting.
· Peoples' mobility trends measured by Google LLC as percentage changes in visitors to specific places relative to a baseline day. A baseline day represents a normal value for that day of the week, and is calculated as the median from the 5-week period January 3 to February 6, 2020. The index is smoothed using a rolling 7-day average. Mobility trends are measured across six broad categories: (1) places of residence; (2) grocery and pharmacy stores, i.e., grocery markets, food warehouses, farmers markets, specialty food shops, drug stores, and pharmacies; (3) places of work; (4) parks, i.e., local parks, national parks, public beaches, marinas, dog parks, plazas, and public gardens; (5) transit stations, i.e., public transport hubs such as subway, bus, and train stations; (6) retail and recreation, i.e., restaurants, cafes, shopping centers, theme parks, museums, libraries, and movie theaters.

Variables collected at daily level (stringency and mobility) were converted into weekly time series by averaging values over the previous week. Furthermore, mobility trends were averaged according to two different types of mobility: workplaces and stations, indicative of workers' mobility; retail and grocery, indicative of consumers' mobility (7). Parks and outdoor spaces were excluded due to extensive volatility in time series influenced by weather and holidays.

Weekly time series were illustrated with the aid of line plots. For each country, the association between stringency index and cumulative excess mortality was investigated by means of vector autoregressive analysis, a multivariate time-series regression of a dependent variable on lags of itself and on lags of the other dependent variable included in the model. More specifically, if the Wald test rejects the null hypothesis that the estimated coefficients on the lagged values of the stringency index are jointly zero, we conclude that the stringency index “Granger-causes” excess mortality, which means that past values of the stringency index may be useful for predicting an increase or decrease in excess mortality (8). As its name implies, Granger causality is not necessarily “true” causality, since it identifies cause-effect relations only by looking at constant conjunctions. The optimal lag order expressing this latency, in weeks, was chosen using the Hannan–Quinn information criterion and sequential likelihood-ratio tests (8). To account for non-stationarity, time series were either first-differenced, detrended, or first-differenced and detrended using the augmented Dickey–Fuller test to check for the presence of a unit root in the autoregressive time series model (9). According to Johansen's multiple-trace test, no cointegration (long-run causality) between time series was present (10).

In a secondary analysis, vector autoregression was replicated to test for the Granger causality of the stringency index on peoples' mobility. All data were analyzed with Stata 17 (11). The significance level was set at 0.05, and all tests were two-sided.

This study involved aggregate data that exist in the public domain, where it is not possible to identify individuals from the information provided. For this reason, this research did not require ethical approval.

## Results

**Figure 1:**
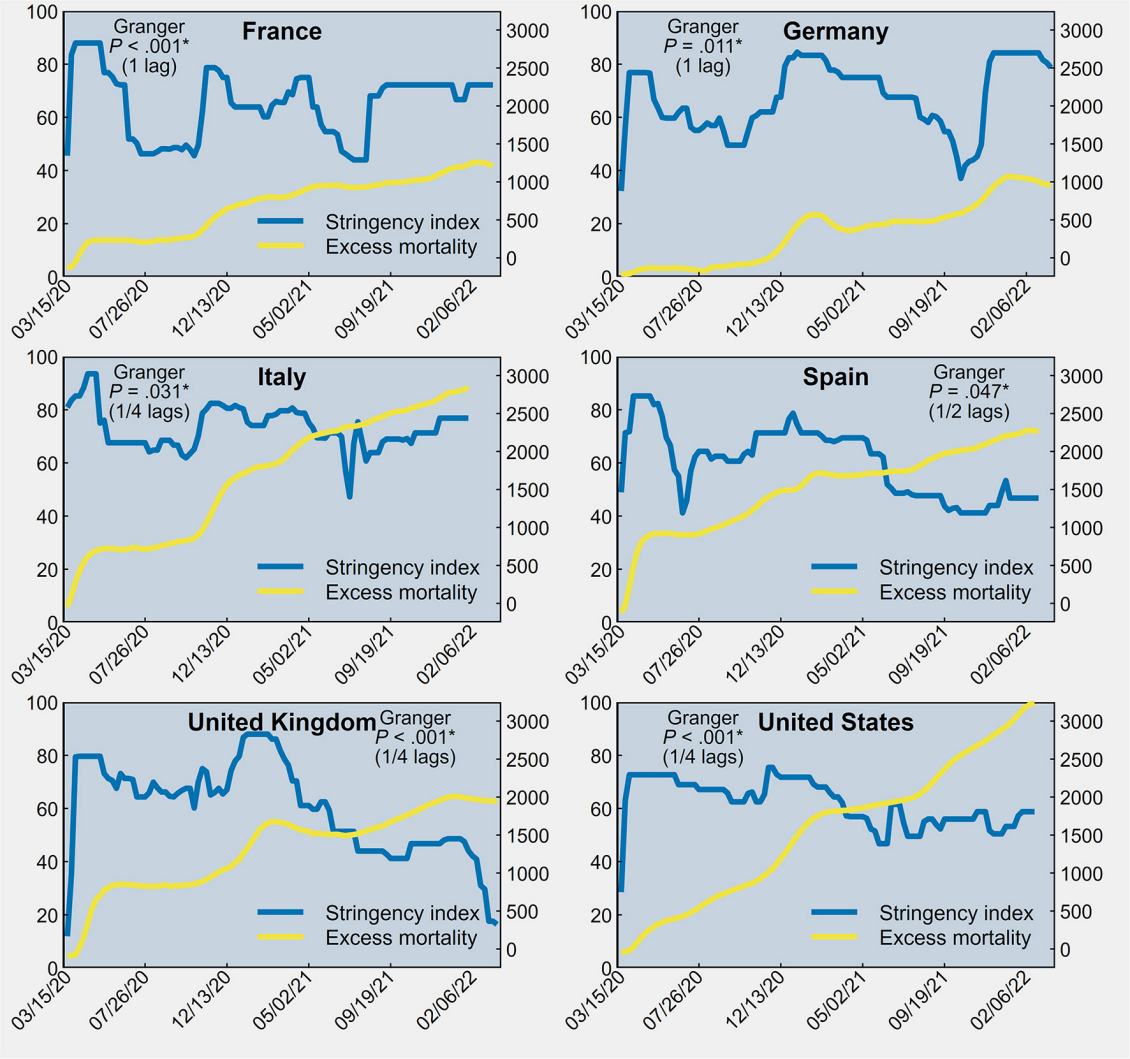
Stringency index (first *y-*axis) and cumulative excess mortality per million population (second *y-*axis) between March 15, 2020 and March 20, 2022 in France, Germany, Italy, Spain, the United Kingdom and the United States of America.

As shown in Figure 1, in each country there was evidence of significant Granger causality of the stringency index on the cumulative rate of excess mortality (*P* ≤ 0.05), with country-specific time delays ranging from 1 to 4 weeks. This means that a decrease in policy stringency was associated with an increase in the overall number of deaths in the following weeks.

As shown in Supplementary Figures S1–S6, in each country there was evidence of significant Granger causality of the stringency index on peoples' mobility, both workers and consumers (*P* ≤ 0.05), with country-specific time delays ranging from 1 to three 3. This means that an increase in policy stringency was associated with an overall decrease in citizens' mobility to workplaces, transit stations, grocery and pharmacy stores, and places of retail and recreation. An exception is mobility to places of work and transport hubs in Italy, which exhibited a borderline significant association with the stringency index (*P* = 0.087), suggesting that Italian workers may have been less heavily influenced by stringency policies than in other countries.

## Discussion

World populations have different ways to endure state restrictions depending on local governments, cultures, and traditions. In countries characterized by a western, democratically imprinted lifestyle, it is possible that both direct and indirect limitations to personal freedom may create greater discontent and intolerance than in countries where the imprint is more authoritarian (12, 13). However, our study suggests that the governments' non-pharmaceutical interventions set up in Western Europe and the US to cope with the pandemic were respected by most citizens and, as a result, contributed to alleviate the strain on health services, irrespective of their type (Beveridge, Bismarck or free-market).

As much as it is understandable the resentment of an individual who experiences a loss of personal freedom, it seems that most citizens accepted unusual and unprecedented constraints for the common good. The governments had to rely upon the co-operation of citizens, since coercion and threats of legal consequences alone are an expensive and ineffective way of securing compliance with the law.

In our opinion, the personal and economic sacrifices of the individual are nothing when compared with the welfare of the community. Given the results obtained by the policy responses to COVID-19 in countering increase in population mortality, we argue that the curtailment of our liberties was not in vain, thanks to the governments' decision to be guided by science and a level of co-operation between citizens and governments that has few equals in history.

## Author Contributions

AC and JL: concept, design, acquisition, analysis, and interpretation of data. DG: critical revision of the manuscript for important intellectual content. JL: statistical analysis and supervision. All authors: drafting of the manuscript. All authors contributed to the article and approved the submitted version.

## Conflict of Interest

The authors declare that the research was conducted in the absence of any commercial or financial relationships that could be construed as a potential conflict of interest.

## Publisher's Note

All claims expressed in this article are solely those of the authors and do not necessarily represent those of their affiliated organizations, or those of the publisher, the editors and the reviewers. Any product that may be evaluated in this article, or claim that may be made by its manufacturer, is not guaranteed or endorsed by the publisher.
