# Supplementary material for: Deaths, Countermeasures, and Obedience: How Countries' Non-pharmaceutical Measures Have Quelled the COVID-19 Death Toll
Source: Front Public Health. 2022 Jun 24;10:934309. doi: 10.3389/fpubh.2022.934309 (PMC9263288; doi:10.3389/fpubh.2022.934309)
Supplement: Supplementary file 1 [file Data_Sheet_1.PDF]

Figure S1. Percentage changes in visitors by category (workers: workplaces and transit stations; consumers: grocery/pharmacy stores and retail/recreation) and stringency index in France between March 15, 2020 and March 20, 2022.

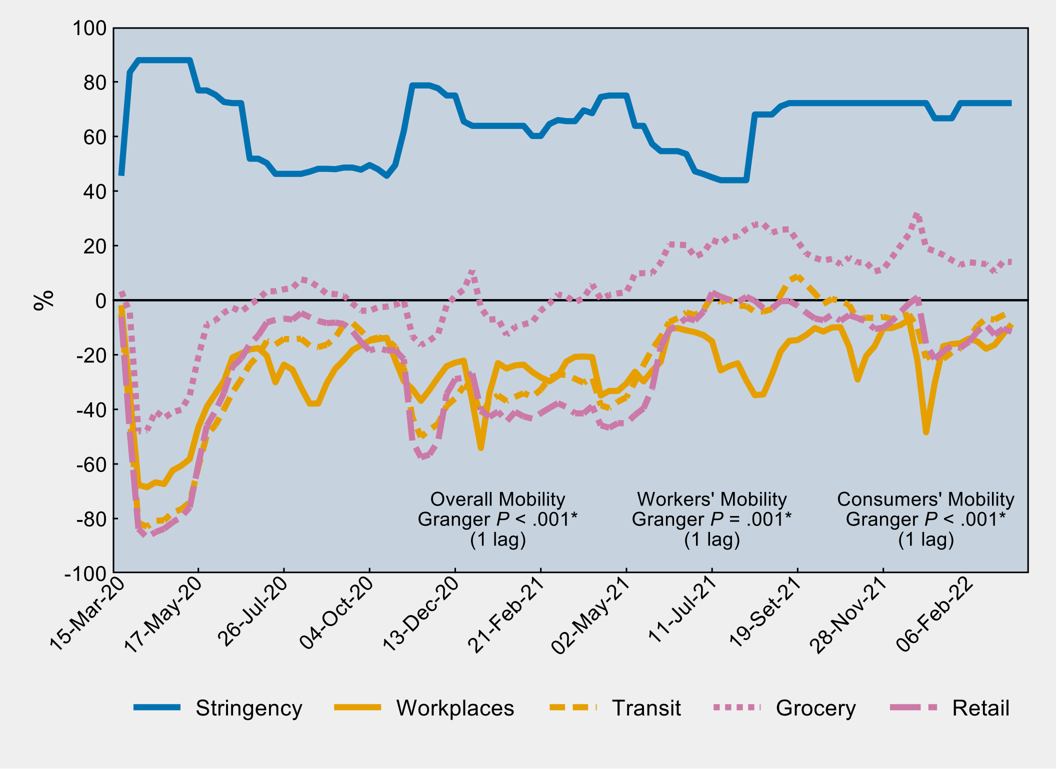

*P-value ≤0.05.
Notes: The stringency index is a composite measure based on nine response indicators including school closures, workplace closures, and travel bans, rescaled to a value from 0 to 100 (100 = strictest). Percentage changes in visitors are calculated relative to a baseline day, i.e., a day representing a normal value for that day of the week. Workplaces include places of work; transit stations include places like public transport hubs such as subway, bus, and train stations; grocery and pharmacy stores include places like grocery markets, food warehouses, farmers markets, specialty food shops, drug stores, and pharmacies; retail and recreation include places like restaurants, cafes, shopping centers, theme parks, museums, libraries, and movie theaters. A significant P-value indicates that the stringency index "Granger-causes" citizens' mobility with a lag length chosen with sequential likelihood-ratio tests.
Sources: Data from Hale et al. (2021) and Google LLC ("Google COVID-19 Community Mobility Reports") via Our World in Data.

Figure S2. Percentage changes in visitors by category (workers: workplaces and transit stations; consumers: grocery/pharmacy stores and retail/recreation) and stringency index in Germany between March 15, 2020 and March 20, 2022.

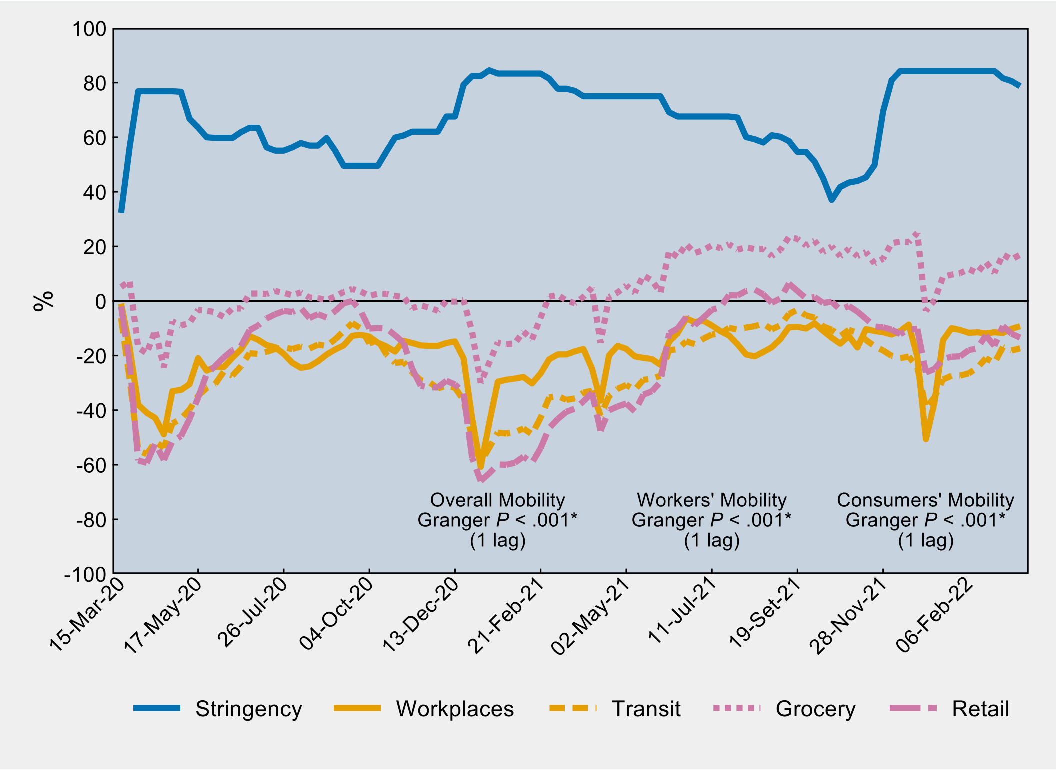

**P*-value ≤0.05.
Notes: The stringency index is a composite measure based on nine response indicators including school closures, workplace closures, and travel bans, rescaled to a value from 0 to 100 (100 = strictest). Percentage changes in visitors are calculated relative to a baseline day, i.e., a day representing a normal value for that day of the week. Workplaces include places of work; transit stations include places like public transport hubs such as subway, bus, and train stations; grocery and pharmacy stores include places like grocery markets, food warehouses, farmers markets, specialty food shops, drug stores, and pharmacies; retail and recreation include places like restaurants, cafes, shopping centers, theme parks, museums, libraries, and movie theaters. A significant *P*-value indicates that the stringency index "Granger-causes" citizens' mobility with a lag length chosen with sequential likelihood-ratio tests.
Sources: Data from Hale et al. (2021) and Google LLC ("Google COVID-19 Community Mobility Reports") via Our World in Data.

Figure S3. Percentage changes in visitors by category (workers: workplaces and transit stations; consumers: grocery/pharmacy stores and retail/recreation) and stringency index in Italy between March 15, 2020 and March 20, 2022.

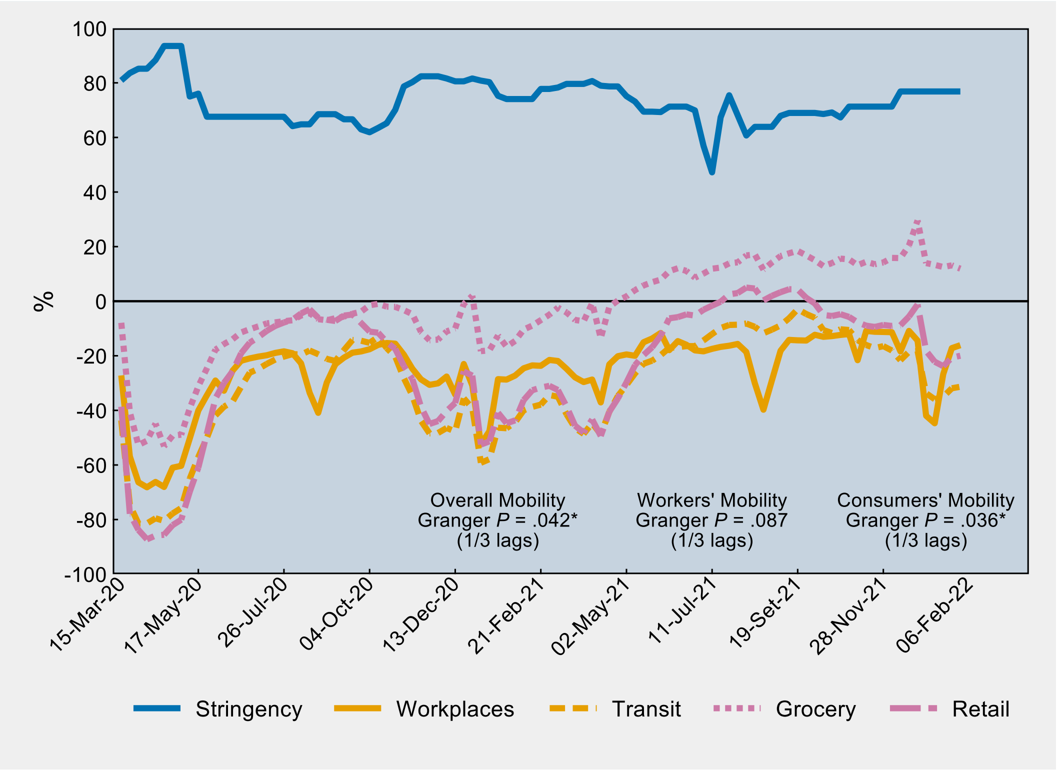

**P*-value ≤0.05.
Notes: The stringency index is a composite measure based on nine response indicators including school closures, workplace closures, and travel bans, rescaled to a value from 0 to 100 (100 = strictest). Percentage changes in visitors are calculated relative to a baseline day, i.e., a day representing a normal value for that day of the week. Workplaces include places of work; transit stations include places like public transport hubs such as subway, bus, and train stations; grocery and pharmacy stores include places like grocery markets, food warehouses, farmers markets, specialty food shops, drug stores, and pharmacies; retail and recreation include places like restaurants, cafes, shopping centers, theme parks, museums, libraries, and movie theaters. A significant *P*-value indicates that the stringency index "Granger-causes" citizens' mobility with a lag length chosen with sequential likelihood-ratio tests.
Sources: Data from Hale et al. (2021) and Google LLC ("Google COVID-19 Community Mobility Reports") via Our World in Data.

Figure S4. Percentage changes in visitors by category (workers: workplaces and transit stations; consumers: grocery/pharmacy stores and retail/recreation) and stringency index in Spain between March 15, 2020 and March 20, 2022.

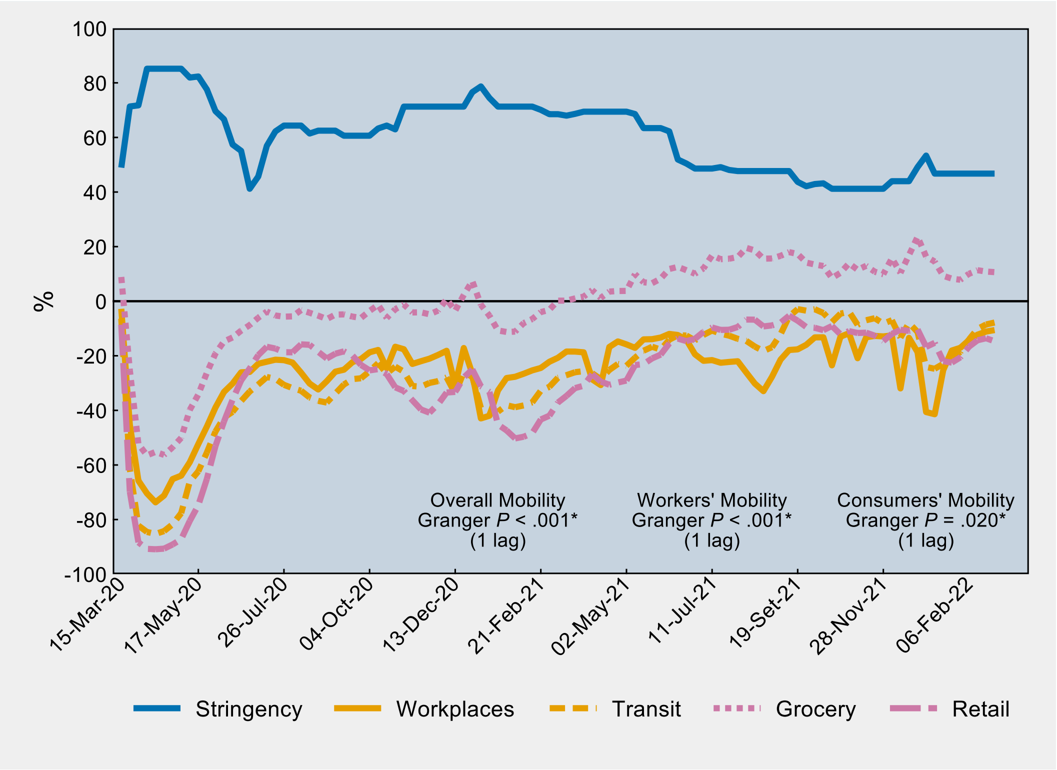

**P*-value ≤0.05.
Notes: The stringency index is a composite measure based on nine response indicators including school closures, workplace closures, and travel bans, rescaled to a value from 0 to 100 (100 = strictest). Percentage changes in visitors are calculated relative to a baseline day, i.e., a day representing a normal value for that day of the week. Workplaces include places of work; transit stations include places like public transport hubs such as subway, bus, and train stations; grocery and pharmacy stores include places like grocery markets, food warehouses, farmers markets, specialty food shops, drug stores, and pharmacies; retail and recreation include places like restaurants, cafes, shopping centers, theme parks, museums, libraries, and movie theaters. A significant *P*-value indicates that the stringency index "Granger-causes" citizens' mobility with a lag length chosen with sequential likelihood-ratio tests.
Sources: Data from Hale et al. (2021) and Google LLC ("Google COVID-19 Community Mobility Reports") via Our World in Data.

Figure S5. Percentage changes in visitors by category (workers: workplaces and transit stations; consumers: grocery/pharmacy stores and retail/recreation) and stringency index in the United Kingdom between March 15, 2020 and March 20, 2022.

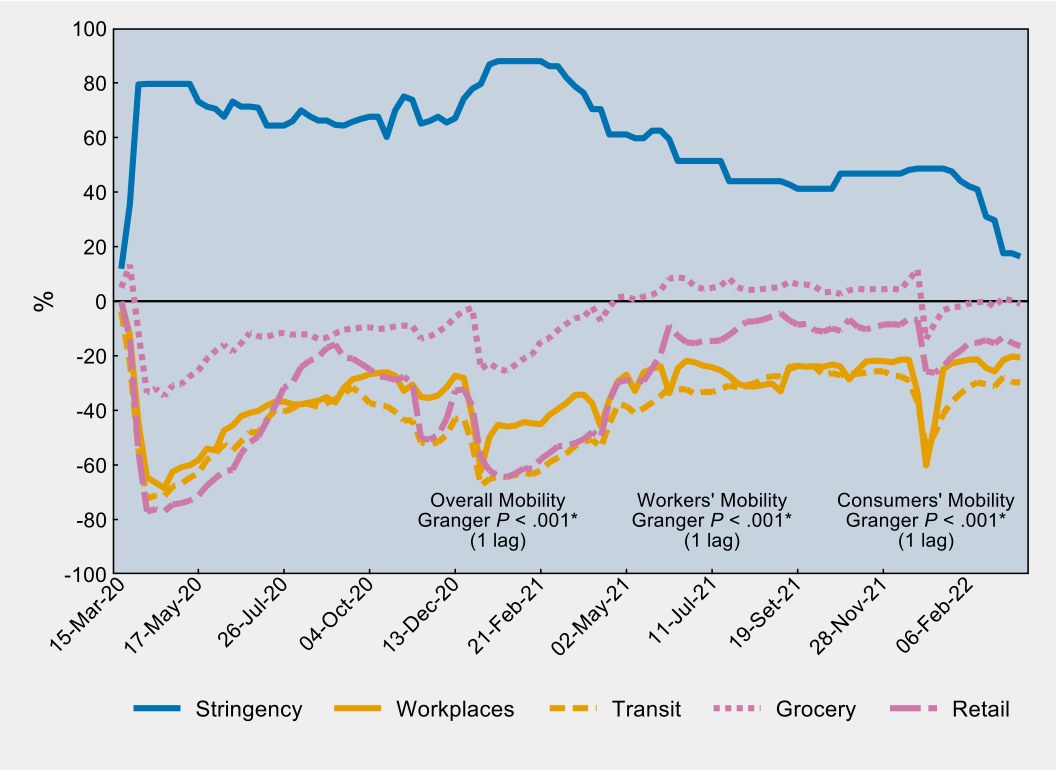

*P-value ≤0.05.
Notes: The stringency index is a composite measure based on nine response indicators including school closures, workplace closures, and travel bans, rescaled to a value from 0 to 100 (100 = strictest). Percentage changes in visitors are calculated relative to a baseline day, i.e., a day representing a normal value for that day of the week. Workplaces include places of work; transit stations include places like public transport hubs such as subway, bus, and train stations; grocery and pharmacy stores include places like grocery markets, food warehouses, farmers markets, specialty food shops, drug stores, and pharmacies; retail and recreation include places like restaurants, cafes, shopping centers, theme parks, museums, libraries, and movie theaters. A significant P-value indicates that the stringency index "Granger-causes" citizens' mobility with a lag length chosen with sequential likelihood-ratio tests.
Sources: Data from Hale et al. (2021) and Google LLC ("Google COVID-19 Community Mobility Reports") via Our World in Data.

Figure S6. Percentage changes in visitors by category (workers: workplaces and transit stations; consumers: grocery/pharmacy stores and retail/recreation) and stringency index in the United States of America between March 15, 2020 and March 20, 2022.

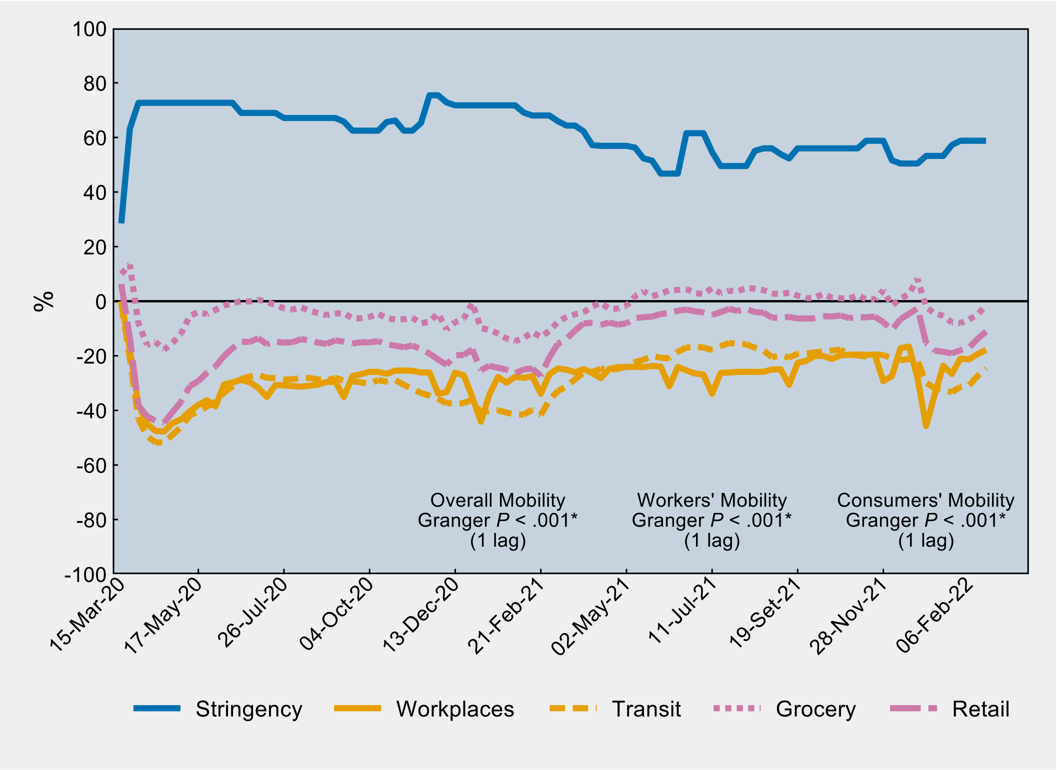

**P*-value ≤0.05.
Notes: The stringency index is a composite measure based on nine response indicators including school closures, workplace closures, and travel bans, rescaled to a value from 0 to 100 (100 = strictest). Percentage changes in visitors are calculated relative to a baseline day, i.e., a day representing a normal value for that day of the week. Workplaces include places of work; transit stations include places like public transport hubs such as subway, bus, and train stations; grocery and pharmacy stores include places like grocery markets, food warehouses, farmers markets, specialty food shops, drug stores, and pharmacies; retail and recreation include places like restaurants, cafes, shopping centers, theme parks, museums, libraries, and movie theaters. A significant *P*-value indicates that the stringency index "Granger-causes" citizens' mobility with a lag length chosen with sequential likelihood-ratio tests.
Sources: Data from Hale et al. (2021) and Google LLC ("Google COVID-19 Community Mobility Reports") via Our World in Data.
